# Satisfaction of patients hospitalised in psychiatric hospitals: a randomised comparison of two psychiatric-specific and one generic satisfaction questionnaires

**DOI:** 10.1186/1472-6963-6-108

**Published:** 2006-08-28

**Authors:** Isabelle Peytremann-Bridevaux, Frédy Scherer, Laurence Peer, Federico Cathieni, Charles Bonsack, Agatta Cléopas, Véronique Kolly, Thomas V Perneger, Bernard Burnand

**Affiliations:** 1 Health Services Research Unit, Institute for Social and Preventive Medicine, University of Lausanne, 17 Bugnon, CH-1005 Lausanne, Switzerland; 2 Health Care Evaluation Unit, Institute for Social and Preventive Medicine, University of Lausanne, 17 Bugnon, CH-1005 Lausanne, Switzerland; 3 Department of Psychiatry, CHUV, University of Lausanne, CH-1000 Lausanne, Switzerland; 4 Quality of Care Service, University Hospitals of Geneva, Micheli-du-Crest 24, CH-1211 Geneva 14, Switzerland; 5 Institute of Social and Preventive Medicine, University of Geneva, University Medical Centre, CH-1211 Geneva 4, Switzerland; 6 Deceased

## Abstract

**Background:**

While there is interest in measuring the satisfaction of patients discharged from psychiatric hospitals, it might be important to determine whether surveys of psychiatric patients should employ generic or psychiatry-specific instruments. The aim of this study was to compare two psychiatric-specific and one generic questionnaires assessing patients' satisfaction after a hospitalisation in a psychiatric hospital.

**Methods:**

We randomised adult patients discharged from two Swiss psychiatric university hospitals between April and September 2004, to receive one of three instruments: the Saphora-Psy questionnaire, the Perceptions of Care survey questionnaire or the Picker Institute questionnaire for acute care hospitals. In addition to the comparison of response rates, completion time, mean number of missing items and mean ceiling effect, we targeted our comparison on patients and asked them to answer ten evaluation questions about the questionnaire they had just completed.

**Results:**

728 out of 1550 eligible patients (47%) participated in the study. Across questionnaires, response rates were similar (Saphora-Psy: 48.5%, Perceptions of Care: 49.9%, Picker: 43.4%; *P *= 0.08), average completion time was lowest for the Perceptions of Care questionnaire (minutes: Saphora-Psy: 17.7, Perceptions of Care: 13.7, Picker: 17.5; *P *= 0.005), the Saphora-Psy questionnaire had the largest mean proportion of missing responses (Saphora-Psy: 7.1%, Perceptions of Care: 2.8%, Picker: 4.0%; *P *< 0.001) and the Perceptions of Care questionnaire showed the highest ceiling effect (Saphora-Psy: 17.1%, Perceptions of Care: 41.9%, Picker: 36.3%; *P *< 0.001). There were no differences in the patients' evaluation of the questionnaires.

**Conclusion:**

Despite differences in the intended target population, content, lay-out and length of questionnaires, none appeared to be obviously better based on our comparison. All three presented advantages and drawbacks and could be used for the satisfaction evaluation of psychiatric inpatients. However, if comparison across medical services or hospitals is desired, using a generic questionnaire might be advantageous.

## Background

Continuous quality improvement, comparison of quality across hospitals, and demands for accountability are some of the reasons that drive hospitals to measure patient satisfaction. An unresolved issue is whether surveys of psychiatric patients should employ generic instruments or questionnaires specifically designed for this healthcare setting. Generic instruments allow comparisons across settings but may lack content validity compared with condition-specific instruments. Several condition-specific instruments for psychiatric inpatients are available, such as the Perceptions of Care questionnaire [1] and the Saphora-Psy questionnaire in France [2].

The choice of the most suitable questionnaire is often left to professionals and patients are rarely consulted. Studies conducted to determine whether a questionnaire would be more suitable than another are rare [3], and none has addressed this issue for psychiatric patients. Several authors have, however, analysed psychiatric inpatient satisfaction [4-10], or developed and validated instruments designed specifically to assess inpatient psychiatric services [1,11-13].

In this study, we compared two psychiatric-specific and one generic questionnaires aimed at evaluating the opinion and satisfaction of psychiatric patients regarding the care they received during hospitalisation. We assessed their characteristics and asked the patients to rate the questionnaire they had just completed.

## Methods

### Study design and setting

Between April and September 2004, we conducted a survey of three satisfaction questionnaires administered at random (computer-generated randomisation) to all eligible patients discharged from psychiatric hospitals of Vaud and Geneva university centres, Switzerland, which are spread over three sites in Vaud and one in Geneva. The randomisation was stratified per site. A random number was assigned to each patient on the monthly discharge list; this list was then sorted by the random number, and consecutive thirds of the patient list received a different questionnaire. On two of the three sites in Vaud (which are smaller hospitals), only two instruments were distributed: either the Perceptions of Care and Picker, or Saphora-Psy and Picker questionnaires. This was motivated by the primary goal of the survey which was to obtain a measure of patient satisfaction in all sites using the Picker Institute questionnaire, already in use there. In Geneva and in the remaining centre in Vaud, all three questionnaires were administered.

### Participants

All psychiatric inpatients aged 18 years and over who had been hospitalised for more than 24 hours were included in the study. Patients treated in psycho-geriatric wards, staying in prison, residing outside Switzerland, deceased during hospitalisation or transferred to another hospital during that stay were excluded. Patients hospitalised multiple times during the survey period were contacted once only. Secondary exclusions, which had been defined a priori, were carried out during data collection (patients who considered themselves or were considered by their proxies to be too sick to complete a questionnaire, who had died after discharge, who did not understand French, or whose address was invalid). Based on the number of patients hospitalised in the psychiatric wards of Geneva and Vaud during April 2003, and on an probable participation rate of 50%, we estimated that the number of patients who could reasonably be included in this study would be 200 per questionnaire.

As for all patient satisfaction surveys and other quality improvement activities, this project was exempted from review by the research ethics committees of the Lausanne and Geneva University Hospitals.

### Data collection

The survey was conducted by mail. The first survey package was sent out 4 to 8 weeks after discharge. A postcard reminder was sent one week after the initial mailing and a full survey package was sent to non-respondents two weeks later, and again approximately 4 to 6 weeks from the initial mailing if no reply was received in-between. The survey package included a cover letter, the randomly assigned questionnaire, a set of evaluation and patients' characteristics items, and a stamped return envelope. The cover letter presented the survey and indicated that participation was voluntary. Confidentiality and anonymity issues were emphasized. In addition, a telephone hotline was set up 3 days a week to answer any questions raised by this survey. A screening question identifying patients too sick to respond, who did not understand French or declined participation, was incorporated on the front page of the questionnaire.

### Questionnaires

We compared three developed satisfaction questionnaires intended to evaluate hospital stays. We selected three candidate instruments that were previously validated, available in French (Picker Institute questionnaire and Saphora-Psy questionnaires) and/or already used for surveys of psychiatric patients in Switzerland (Perceptions of Care questionnaire and Picker Institute questionnaire). Two were specific to adult psychiatric inpatients (Perceptions of Care survey [1], Saphora-Psy [2]), while the third one was designed for adult acute care inpatients (Picker Institute questionnaire for acute inpatient somatic care) [14-16]. Both the Saphora-Psy and the Picker Institute questionnaire were based on report, experience whereas the Saphora-Psy was mainly a satisfaction questionnaire, per-se. While the Picker Institute and the Saphora-Psy questionnaires were developed with the use of qualitative methods aimed to explore patients' needs and concerns, the Perceptions of Care questionnaire was developed by selecting items related to specific domains, from previous work and literature review. In addition, the Saphora-Psy questionnaire was developed in French and a French translation was available for the Picker Institute questionnaire. Our group prepared a French translation of the Perceptions of Care questionnaire. We made six parallel translations in French followed by a consensus version obtained after discussion, which was back translated in English by an independent person of English mother tongue. The questionnaires are extensively described in Table 1.

At the end of each satisfaction questionnaire, the patient was asked to record the time needed to complete it, and to reply to ten questions evaluating the questionnaire. A few patients' characteristics were also recorded, whereas patient's age and sex were retrieved from administrative charts.

### Main outcome measures

The three satisfaction questionnaires were compared in terms of four characteristics and patient evaluation ratings. Characteristics included the response rate, the completion time, the mean proportion of missing items and the mean proportion of responses which gave the highest available rating (ceiling effect). These outcomes were selected because they, respectively, reflect: the acceptability and relevance to patients of the questionnaire as a whole, the respondent's burden, the adequacy of the items and ease of finding a suitable answer, and the ability to discriminate a high level of performance.

The ten evaluation ratings assessed the patient's opinion about the questionnaire just completed: difficulty to fill in the questionnaire, clarity of question formulation, importance of the questions addressed, ease of assessment of patients' concerns, missing of important questions, ease of finding suitable answers, length of questionnaire, lay-out, appropriateness of wording, general opinion about the questionnaire

### Statistical analysis

We used chi-squared tests for comparisons of categorical or dichotomous variables, oneway ANOVA for comparisons of means of continuous variables, and Kruskall-Wallis for non-normal variables.

For the calculation of i) the mean proportion of missing values and ii) the mean proportion of responses with the highest available rating (ceiling effect), the denominator corresponded to the total number of valid answers. For i) all questions except open-ended questions were considered valid; for ii) in addition, questions for which a yes/no answer was available were not counted as valid. Thus, we performed the calculation of the mean proportion of missing values on 34 out of 36 questions for the Saphora-Psy questionnaire, 18 out of 21 questions for Perceptions of Care questionnaire and all 50 questions for the Picker questionnaire. To compute the proportion of responses corresponding to the highest available rating, we used 31 out of 36 questions for the Saphora-Psy questionnaire, 13 out of 21 questions for the Perceptions of Care questionnaire and 35 out of 50 questions for the Picker questionnaire.

## Results

A total of 1764 survey questionnaires were mailed to patients. After three complete mailings, 214 patients were found to be ineligible because of invalid address, patient death, patient too sick or inability to understand French. 728 answered the questionnaire, resulting in an overall participation rate of 47%.

The mean age of respondents was 40 years (SD 12.5); 59% were women, 71% were Swiss and the majority received compulsory or vocational training (63%). Except for depressive symptoms, the three randomised groups of patients were not significantly different (Table 2).

### Characteristics of the questionnaires

Table 3 shows a comparison of the characteristics of the questionnaires. The response rates varied from 43% to 50%. Mean completion time was significantly lowest for the Perceptions of Care questionnaire. The mean proportion of missing values was highest for the Saphora-Psy questionnaire, which, however, showed the lowest mean ceiling effect. The Perceptions of Care questionnaire had the lowest mean proportion of missing values but the highest proportion of ceiling effect. The Picker Institute questionnaire showed intermediate results.

### Evaluation ratings

Although the three instruments differed and were not all primarily targeted towards psychiatric inpatients, patients rated them similarly (Table 4). There was often a statistical bivariate relationship between the ten evaluation questions, which did not appear to be completely independent from each other (median Spearman correlation coefficient 0.256 (range -0.013 to 0.502), however. In addition, 4 of the 21 Perceptions of Care questions were indicated as being unclear or difficult to understand by more than 10 respondents.

## Discussion

Our findings show that, despite differences in design and purposes, content, lay-out, length and initial target population of the three questionnaires, none appeared to be obviously better than the others, both when examining their characteristics and the patients' assessments. All three appeared to have advantages and drawbacks.

Response rates did not differ across questionnaires, regardless of their lengths. In previous studies, higher response rates have not been consistently associated with shorter questionnaires [3,17,18]. It is possible that the patients' motivation and interest in the topic may be more important than the actual length of the questionnaire.

The high proportion of missing responses found in the Saphora-Psy questionnaire could be at least partly explained by the presence of numerous questions starting with a conditional clause, such as "If you have had ...", that does not offer answer options for patients who do not meet this initial criterion. In addition, Saphora-Psy is supposed to be completed at the end of the hospitalisation, not after discharge. This is however also true for the Perceptions of Care questionnaire, which showed the lowest mean proportion of missing answers. The way questions and answers options are organised in the Saphora-Psy questionnaire may thus not be optimal. Developed for acute care inpatients, the Picker instrument includes a set of questions related to pain and surgery. Interestingly, these items did not get a high proportion of missing data, although pain may not have the same meaning for acute care and psychiatric patients, and surgery is rarely used during psychiatric hospitalisation. This may reflect the appropriateness of skip patterns.

Not surprisingly, the Perceptions of Care questionnaire, which has the lowest mean number of response categories per question, showed the highest ceiling effects. This questionnaire may therefore be less sensitive to changes and less able to discriminate at the high end of the satisfaction spectrum.

Because disease-specific health status or quality-of-life questionnaire often perform differently from generic instruments [19,20], we were surprised not to detect differences between the psychiatry-specific and generic questionnaires. Examination of their contents suggests that the two psychiatry-specific questionnaires did not differ much from the generic instrument. An alternative hypothesis is that patients hospitalised in mental health facilities have the same basic needs and expectations as any other patients. Another possibility is that we did not consider all the discriminative characteristics allowing to detect true differences between those three questionnaires.

The randomised allocation of questionnaires, the use of three methodically developed satisfaction instruments and the relatively high number of psychiatric inpatients included were important strengths of this study. However, our study does have limitations. First, the participation rate was relatively low [21] but similar to those reported in other satisfaction studies of psychiatric patients, who are less likely to respond to questionnaires compared to other patients [8,22]. The effect of selection bias on the measures of interest is unpredictable; however, because the participation rate was similar for all three instruments, the comparison of the questionnaires are likely to be internally valid. Second, the sample size could have prevented the detection of true differences between questionnaires. Third, patients did not make comparative judgements on the three questionnaires, which may have given different evaluation results. They may also not be the best judges to objectively assess questionnaires. In addition, there was a correlation between the ten evaluation questions assessing the patient's opinion about the questionnaires, with a between item correlation which was moderate on average. Even though this statistical association could suggest that these outcome measures should not be considered separately, we analysed each question separately, because we were interested in each outcome variable as such. Moreover, given the lack of differences among the three questionnaires, it would be unlikely that a composite index would show differences. Fourth, we centred our study on patients and did not assess other healthcare stakeholders opinion. Indeed, for quality improvement purposes, their opinion about the usefulness of selected satisfaction questionnaire might be of interest. Finally, we evaluated only three out of several satisfaction questionnaires.

As we did not address all possible aspects related to the selection of a satisfaction questionnaire, further research would be needed to assess, for example, the opinion of other healthcare stakeholders, whether questionnaires perform equally with all types of patients hospitalized in psychiatric wards, and which questionnaire would be best for a specific situation.

## Conclusion

Our results suggest that all three satisfaction questionnaires, one generic and two psychiatry-specific instruments, presented advantages and drawbacks, and obtained similar results in patients' evaluation ratings for the features we examined, despite differences in their design and purposes, intended target population, content, lay-out and length. Accordingly, they could all be used for the satisfaction evaluation of patients admitted in psychiatric hospitals. However, other criteria might be considered to decide which questionnaire is the most suitable in a given context. For instance, if comparison across medical services or hospitals is the main objective, at least on a set of items, and practical constraints have to be taken into account, using a single generic questionnaire might be advantageous.

## Competing interests

The author(s) declare that they have no competing interests.

## Authors' contributions

IPB analysed and interpreted the data, and drafted the manuscript. FC, FS, LP, and VK participated in the design of the study, helped in the acquisition and analysis of the data, and to draft the manuscript. CB participated in the conception and design of the study and helped to draft the manuscript. AC (deceased) participated in the design of the study and helped in the acquisition of the data. BB and TVP conceived the study, participated in its design and coordination, helped in the analysis of the data and critically revised the manuscript.

All authors read and approved the final version of the article

## Pre-publication history

The pre-publication history for this paper can be accessed here:



## Figures and Tables

**Table 1:** Characteristics of three inpatient satisfaction questionnaires

	**Saphora-Psy**	**Perceptions of Care survey**	**Picker Institute**
**Development**	Comité de Coordination de l'Evaluation Clinique et de la Qualité en Aquitaine (CCECQA), France	Harvard Medical School, Department of Psychiatry, Mc Lean Hospital, Boston, MA, USA	Picker Institute, Boston, MA, USA (currently Oxford, UK)
			
**French translation**	Initially developed in French	French translation made by our research group (see method section)	Version translated by A. Leplège and A. Giraud, Public Hospitals of Paris, France (not the official version)
			
**Type of instrument**	Satisfaction per-se (mainly)	Report, experience	Report, experience
			
**Version and number of items**	Adult psychiatric inpatient version, 34 items (of which 1 to determine skip pattern) and 2 open-ended questions	Adult psychiatric inpatient version, 18 items and 1 open-ended question	Adult somatic inpatient version, 50 items (of which 7 to determine skip patterns) and 1 open-ended question
			
**Administration of the questionnaire**	Before discharge (in-hospital)	Before discharge (in-hospital)	After discharge (out of hospital)
			
**Layout**	Response options in row below question	Questions left, response options right, on same row	Response options in separate rows below question (page divided into 2 columns)
			
**Sections (number of Items/section)**	Admission (4)	No specific sections	Emergency room (3)
	Nursing staff (9)		Admission (4)
	Physicians (3)		Physicians (7)
	Patients (2)		Nurses (6)
	Services (14)		Hospital staff (10)
	Discharge (2)		Pain control (7)
	General impression (2)		Surgery (5)
			Discharge (5)
			General impression (3)
			
**Domains assessed**	- Continuity of care	- Information received	- Access
	- Nursing care	- Interpersonal aspects of care	- Respect for patients' values, preferences and expressed needs
	- Medical care	- Continuity-coordination of care	- Coordination and integration of care
	- Relation with other patients	- Global evaluation of care	- Information, communication and education
	- Services and performance		- Physical comfort
	- Summary score		- Emotional support and alleviation of fear and anxiety
			- Involvement of family and friends
			- Transition and continuity
			
**Response scales**	All except 3 items rated on a 5-point scale, 2 items rated on a 3-point scale (yes/no/don't know), 1 yes/no item	Most items rated on a 4-point scale, 4 items on a 3-point scale, 4 yes/no items, 1 item on a 10-point scale	Most items rated on a 3 or 5-point scale, 8 items on a 4-point scale, 1 item on time scale
			
**Example of question and its response options**	"What do you think about the communication (information, explanations) with the staff about your healthcare (treatments, physicians' visits, ...)?" *excellent/verygood/good/poor/very poor*	"Were you involved as much as you wanted in decisions about your treatment?" *never/sometimes/usually/always*	"When you had important questions to ask a doctors, did you get answers you could understand?" *yes, always/yes, sometimes/no/didn't have questions*
			
**Open-ended questions**	"Do you have remarks or comments? Do not hesitate to write them on this page"	"Is there anything else you would like to tell us about your care?"	"If you could change one thing about the hospital, what would it be?"
			
**Reference**	Public use of instrument after contact and authorization; see . Required note of copyright. Contact: secretariat@ccecqa.asso.fr	Original English version in public domain. Eisen SV & al. Assessing consumer perceptions of inpatients psychiatric treatment: the perception of care survey. Jt Comm J Qual Improv. 2002;28:510-26	Instrument not in public domain; see Contact: info@pickereurope.ac.uk

**Table 2:** Characteristics of respondents according to the study group (as defined by the randomly assigned questionnaire), n and (%) -except when specified differently

	**Saphora-Psy**		**Perceptions of Care**		**Picker**		P-value*
	**(n = 226)**		**(n = 240)**		**(n = 262)**		
	**n**	**(%)**	**n**	**(%)**	**n**	**(%)**	
							
Male	94	(41.6)	94	(39.2)	112	(42.8)	0.71
							
Age, mean (SD)	40.9	(13.3)	38.4	(12.1)	40.6	(12.0)	0.05
							
Nationality							
Swiss	159	(70.4)	162	(67.5)	196	(74.8)	0.12
*European*	51	(22.6)	49	(20.4)	49	(18.7)	
*Other*	16	(7.0)	29	(12.1)	17	(6.5)	
							
Education							
*Compulsory or vocational*	135	(64.9)	131	(59.3)	168	(65.9)	0.29
							
Health Status							
*Excellent or very good*	36	(17.4)	44	(19.6)	36	(14.6)	0.12
*Good*	91	(44.0)	117	(52.0)	117	(47.6)	
*Fair or poor*	80	(38.7)	64	(28.4)	93	(37.8)	
							
Change in health since first day of admission							
*Better (much or somewhat)*	143	(67.8)	173	(76.6)	184	(73.0)	0.37
*About the same*	52	(24.6)	41	(18.1)	52	(20.6)	
*Worse (much or somewhat)*	16	(7.6)	12	(5.3)	16	(6.4)	
							
In the past month, felt downhearted and blue							
*All, most, a good bit of the time*	124	(58.8)	99	(43.0)	127	(50.2)	0.02
*Some or a little of the time*	73	(34.6)	106	(46.1)	106	(41.9)	
*Never*	14	(6.6)	25	(10.9)	20	(7.9)	
							
During the past 12 months, how many times were you hospitalised, mean (SD)	2	(1.5)	2	(1.7)	1.9	(1.4)	0.90

* P-*values *from chi-squared tests (categorical variables) and oneway ANOVA (age and times hospitalised)

**Table 3:** Measurement characteristics of three inpatient satisfaction questionnaires used in psychiatric hospitals

	**Saphora-Psy**	**Perceptions of Care**	**Picker**	**P-*value***
	**(n = 226)**	**(n = 240)**	**(n = 262)**	
Response rate	48.5 %	49.9 %	43.5 %	0.08
				
Completion time in minutes, mean (SD)	17.7 (12.6)	13.7 (11.0)	17.5 (13.6)	0.005
				
Mean proportion of missing responses per question	7.1 %	2.8 %	4.0 %	0.000
				
Mean proportion of highest responses per question (ceiling effect)	17.1 %	41.9 %	36.3 %	0.000

**Table 4:** Patients' evaluation of inpatient satisfaction questionnaires used in psychiatric hospitals, -mean (standard deviation)

	**Saphora-Psy**		**Perceptions of Care**		**Picker**		P-value*
	**(n = 226)**		**(n = 240)**		**(n = 262)**		
« Was the questionnaire easy or difficult to fill in? »	1.55	(0.67)	1.67	(0.74)	1.63	(0.65)	0.25
[1 = very easy/2 = easy/3 = difficult/4 = very difficult]							
							
« Were the questions that addressed your hospital stay clearly formulated? »	1.61	(0.73)	1.52	(0.74)	1.56	(0.7)	0.25
[1 = yes, all questions/2 = yes, most questions/half of the questions/3 = only some questions/4 = none]							
							
« Do the questions address aspects of your hospital stay that are important to you? »	1.98	(0.93)	2.06	(0.97)	2.09	(0.97)	053
[1 = yes, all questions/2 = yes, most questions/3 = half of the questions/4 = yes some questions/5 = none]							
							
« Do the questions concern aspects that you can easily assess? »	1.83	(0.79)	1.89	(0.9)	1.84	(0.81)	0.96
[1 = yes, all questions/2 = yes, most questions/3 = half of the questions/4 = yes some questions/5 = none]							
							
« Are there important questions missing ? »	2.51	(0.64)	2.44	(0.63)	2.44	(0.65)	0.43
[1 = yes, many are missing/2 = yes, some are missing/3 = none are missing]							
							
« Was it easy to find a suitable answer? »	1.96	(0.9)	1.90	(0.95)	1.85	(0.78)	0.45
[1 = yes, all questions/2 = yes, most questions/3 = half of the questions/4 = yes some questions/5 = none]							
							
« How do you considerthe length of the questionnaire ? »	1.36	(0.6)	1.25	(0.54)	1.36	(0.56)	0.11
[1 = appropriate/2 = somewhat too long/3 = really too long]							
							
« Is the lay-out of the questionnaire clear and legible ? »	1.18	(0.41)	1.16	(0.42)	1.20	(0.43)	0.72
[1 = very clear and legible/2 = clear and legible enough/3 = not clear or not legible enough]							
							
« Were there terms used in this questionnaire that you found inappropriate or shocking? »	2.92	(0.34)	2.86	(0.46)	2.93	(0.27)	0.65
[1 = yes, many terms/2 = yes, some terms/3 = no, none of the terms]							
							
« In your opinion, the questionnaire that you filled in is ? »	2.44	(0.89)	2.56	(0.84)	2.60	(0.82)	0.11
[1 = excellent/2 = very good/3 = good/4 = fair/5 = poor]							

* P-*values *from Kruskal-Wallis test
